# Spending by the Centers for Medicare & Medicaid Services Before and After Confirmation of Benefit for Drugs Granted US Food and Drug Administration Accelerated Approval, 2012 to 2017

**DOI:** 10.1001/jamahealthforum.2022.1158

**Published:** 2022-05-27

**Authors:** Joshua J. Skydel, Alexander C. Egilman, Joshua D. Wallach, Reshma Ramachandran, Ravi Gupta, Joseph S. Ross

**Affiliations:** 1 Dartmouth−Hitchcock Medical Center, Lebanon, New Hampshire; 2 Program on Regulation, Therapeutics, and Law, Division of Pharmacoepidemiology and Pharmacoeconomics, Department of Medicine, Brigham and Women’s Hospital and Harvard Medical School, Boston, Massachusetts; 3 Department of Environmental Health Sciences, Yale School of Public Health, New Haven, Connecticut; 4 National Clinician Scholars Program, Department of Internal Medicine, Yale School of Medicine, New Haven, Connecticut; 5 Veterans Affairs Connecticut Healthcare System and Yale University, West Haven; 6 National Clinician Scholars Program, Corporal Michael J. Crescenz Veterans Affairs Medical Center, Philadelphia, Pennsylvania; 7 Leonard Davis Institute of Health Economics, University of Pennsylvania, Philadelphia; 8 Section of General Medicine, Department of Internal Medicine, Yale School of Medicine, New Haven, Connecticut; 9 Department of Health Policy and Management, Yale School of Public Health, New Haven, Connecticut; 10 Center for Outcomes Research and Evaluation, Yale−New Haven Hospital, New Haven, Connecticut

## Abstract

**Importance:**

Accelerated approval by the US Food and Drug Administration (FDA) grants market authorization for drugs based on clinical trials using surrogate end points likely to anticipate a clinical benefit. The FDA requires postapproval trials to confirm benefit, after which the accelerated approval is converted to a standard approval or is withdrawn. However, trials frequently fail to assess clinical benefit, and expenditure for these drugs may be substantial.

**Objective:**

To evaluate spending by the Centers for Medicare & Medicaid Services (CMS) on drugs granted FDA accelerated approval before and after confirmation of benefit.

**Design and Setting:**

This was a cross-sectional study of CMS spending on drugs granted FDA accelerated approval for original indications from 2012 to 2017, with follow-up through 2020. Using data from the Drugs@FDA database and a validated methodology, the characteristics of new drugs granted FDA accelerated approval were identified and analyzed, including indication area, type of drug, type of confirmatory trial end point, orphan designation, number of supplemental indications, and conversion status.

**Main Outcomes and Measures:**

Overall and annualized spending by Medicare Parts B and D and Medicaid from 2012 to 2020, before and after conversion to standard approval. Analyses were conducted from June 30, 2021, to March 21, 2022.

**Results:**

From 2012 to 2017, the FDA granted accelerated approval to 38 drugs for 42 original indications. Through 2020, CMS spending for these drugs was $67.9 billion (median [IQR], $329.3 million [$54.4 million-$1.6 billion]). For 22 drugs (58%) that were converted to standard approval on the basis of clinical trial results, annualized spending increased substantially after conversion ($35.0 million vs $199.0 million), and spending after conversion accounted for $51.0 billion (75%) of overall spending. However, only 6 conversions (27%) of the 22 were supported by confirmatory trials evaluating clinical outcomes as primary end points. Drugs evaluated using surrogate end points accounted for $40.3 billion (59%) of CMS spending.

**Conclusions and Relevance:**

The findings of this cross-sectional study indicate that most of the drugs granted FDA accelerated approval for original indications from 2012 to 2017 lacked confirmatory trials evaluating clinical outcomes to support conversion to standard approval. Automatic coverage mandates produced substantial CMS spending for drugs with unproven clinical benefits.

## Key Points

**Question:**

How much do the Centers for Medicare & Medicaid (CMS) spend on drugs granted accelerated approval by the US Food and Drug Administration (FDA), before and after confirmation of the drug’s benefit?

**Findings:**

This cross-sectional study of 38 drugs granted FDA accelerated approval from 2012 to 2017 found that CMS spent nearly $68 billion through 2020, including $51 billion (75%) after conversion to standard approval following confirmatory trial results. However, 59% of the spending was for drugs with confirmatory trials that evaluated surrogate end points.

**Meaning:**

The findings of this study indicate that many drugs granted FDA accelerated approval lack evidence of clinical benefit, even after being converted to standard approval, yet they account for substantial CMS spending.

## Teaser

This cross-sectional study evaluates spending by the Centers for Medicare & Medicaid on drugs granted FDA accelerated approval before and after confirmation of benefit from 2012 to 2017 with follow-up through 2020.

## Introduction

The accelerated approval pathway permits the US Food and Drug Administration (FDA) to grant market authorization to drugs and biologics intended to treat serious conditions (eg, cancer) and address unmet medical needs, based on trials using surrogate end points that are “reasonably likely” to anticipate a clinical benefit.^1^ After accelerated approval, sponsors are required to complete postapproval trials to confirm clinical benefit.^2^ Based on the results of confirmatory trials, the FDA may convert the accelerated approval to a standard approval or withdraw the authorization.^1^ Use of accelerated approval has increased since the pathway was established in 1992,^3,4^ with more than 250 therapeutic indications granted accelerated approval to date.^5^ Although accelerated approval facilitates earlier access to drugs requiring prolonged study periods to confirm clinical benefit, evidentiary gaps often persist in the postmarket period.^6^

The FDA allows drug sponsors an extensive period of time to complete confirmatory trials after an accelerated approval is granted, often more than twice as long as is required to evaluate primary end points.^7,8^ Even so, confirmatory trials are frequently delayed and may have limitations similar to those of preapproval studies, including continued use of surrogate end points to evaluate efficacy.^9,10,11^ Consequently, clinical benefit may remain uncertain for years after accelerated approval.^12^ During this period, drugs are integrated into clinical practice and used for unapproved indications,^13^ broadening their use before evidence of efficacy for original indication is confirmed. Moreover, confirmatory trials frequently do not verify clinical benefit; for example, only 1 in 5 cancer drugs that were granted accelerated approval improved overall survival in confirmatory trials.^14^ However, the FDA has rarely withdrawn indications when clinical benefit was not confirmed, and drugs continue to be recommended in clinical guidelines, even after negative confirmatory trials.^15,16^ Lack of incentives for sponsors to conduct trials in a timely manner, evidentiary burdens for FDA action, and resistance to withdrawals from patients and physicians have been posited as barriers to enforcement.^15,17,18^

Although intended to foster earlier access to promising drugs, evidence suggests that the accelerated approval pathway is not associated with added value relative to existing therapeutic options.^19^ Accelerated approval has also been shown to complicate decision-making for physicians and patients^20,21,22^ and for payers, eg, the Centers for Medicare & Medicaid Services (CMS), when determining whether to cover drugs with unknown benefits.^23^ Medicare provides insurance coverage to 63 million individuals (most of whom are 65 years of age or older) and Medicaid covers 74 million individuals with low income in the US.^24^ Drugs granted accelerated approval represent up to 9% of annual Medicaid spending, despite accounting for less than 1% of use.^25^ For Medicare, these drugs accounted for 16% and 2.5% of 2019 spending by Parts B and D, respectively, and annual spending has increased steadily since 2015—driven largely by a subset of high-grossing drugs.^26^ However, it is not known whether confirmation of benefit, particularly via trials evaluating clinical outcomes, is associated with changes in spending. Therefore, we estimated CMS spending on new drugs granted FDA accelerated approval from 2012 to 2017, before and after conversion to standard approval or withdrawal, and assessed whether evaluation of clinical benefit was associated with differences in spending.

## Methods

This study used publicly available data and did not require institutional review or informed consent. The study adhered to the Strengthening the Reporting of Observational Studies in Epidemiology (STROBE) reporting guideline for cross-sectional studies.

### Identifying Drugs Granted Accelerated Approval

Using the Drugs@FDA database^27^ and a previously described methodology,^28^ we identified all therapeutic applications that were granted FDA accelerated approval from 2012 to 2017. The start date, January 1, 2012, was chosen because standardized CMS spending data first became available that year; and the cutoff date, December 31, 2017, allowed a minimum of 36 months for conversion from accelerated to standard approval. Conversion indicated that the FDA had received evidence to confirm a benefit. Spending data were available through 2020.

We limited the sample to drugs granted FDA accelerated approval for original indications. Information was collected on the type of drug (small molecule or biologic), its original approved indication (cancer, including hematologic disease, vs noncancer per the World Health Organization’s Anatomical Therapeutic Chemical Classification system^29^), and whether the original indication received orphan drug designation. Lastly, we identified dates of supplemental indication approvals for each drug.

### Assessing Confirmatory Trial and Accelerated Approval Status

Using FDA approval letters, we identified confirmatory trials for each drug and FDA deadlines for trial completion and submission of trial reports. Then we identified corresponding study registrations from ClinicalTrials.gov^30^ and published trial results using previously described methods.^28,31^ For each trial, we abstracted primary completion date (ie, date of final data acquisition for primary outcomes, per published trial results or from ClinicalTrials.gov if unpublished) and type of primary efficacy end point evaluated (clinical outcome or surrogate end point, using previously described methods^32^). Confirmatory trials were considered positive if the results for the primary efficacy end point were statistically significant (*P* < .05) in favor of the drug and negative if not. We abstracted single-arm study results from author’s statements; for example, a conclusion that a drug “provides a durable benefit” was considered a positive trial outcome.

Using FDA-reported data through December 31, 2020,^5^ we identified dates of a drug’s accelerated approval and conversion to standard approval or withdrawal and calculated the elapsed time in months. We also calculated time from confirmatory trial completion to conversion or withdrawal, time after conversion through 2020, and time from accelerated approval to first supplemental indication approval for drugs.

### Estimating CMS Spending

We used prescription drug event data, which are publicly available from the CMS Drug Spending Dashboards,^33^ to assess therapeutic spending after accelerated approval. Under fee-for-service or traditional Medicare, Part B provides coverage for drugs administered in outpatient health care settings, whereas Part D provides coverage for self-administered drugs. Medicaid provides prescription drug coverage to low-income individuals in the US.^24^ Standardized aggregate spending data were available for Medicare Parts B and D and Medicaid for 2012 through 2020.

For each drug, we calculated CMS spending from the date of accelerated approval through 2020. For each converted drug, we calculated overall and annualized spending before and after conversion, prorated to the month of conversion. We also calculated spending for unconverted drugs, and for drugs with confirmatory trials failing to confirm benefit. Spending was adjusted for inflation to 2020 US dollars, based on the US Consumer Price Index for Medical Care.^34^

We estimated sponsor-provided rebates using an adaptation of previously described methods.^35,36^ Using pricing data from SSR Health,^37^ we adjusted overall spending for estimated annual drug rebates, and for average therapeutic class rebates when individual rates were not available. When data were not available for a specific year, the drug’s rebate rate for the next closest year was used. For 8 drugs without individual or class rebate data available, we assumed the most recently reported overall rebate rate for nongeneric drugs covered under Medicare Part D, 17.5%, and the minimum basic Medicaid rebate of 23.1%.^38^ Rebates are not provided for drugs covered by Medicare Part B.

### Statistical Analyses

We summarized the characteristics of new drugs granted FDA accelerated approval from 2012 to 2017, including indication area, type of drug, type of confirmatory trial end point, orphan designation, and conversion status, using descriptive statistics. Spending by CMS was stratified by these therapeutic characteristics. We performed sensitivity analyses to estimate spending before approval of supplemental indications, and to adjust for the number of supplemental indications approved each year. We evaluated associations between therapeutic characteristics and conversion status using Fisher exact tests. Statistical tests were 2-sided with significance defined as *P* ≤ .05. Analyses were conducted from June 30, 2021, to March 21, 2022, using R, version 4.1.1 (R Foundation for Statistical Computing).

## Results

### Drugs Granted Accelerated Approval

From 2012 to 2017, the FDA granted accelerated approval to 38 drugs with 42 original indications, 28 (74%) of which were indicated to treat cancer (Table 1). Orphan drug designation was granted for nearly all (35 drugs; 92%).

**Table 1.** Drugs Granted FDA Accelerated Approval for Original Indications and Converted to Standard Approval, 2012 to 2017

	Drugs, No. (%)			*P* value
	Total	Converted^a^	Not converted	
All drugs	38	22 (58)	16 (42)	NA
Approval year				.41
2012	6	4 (67)	2 (33)	
2013	2	2 (100)	0 (0)	
2014	8	5 (63)	3 (38)	
2015	8	6 (75)	2 (25)	
2016	7	2 (29)	5 (71)	
2017	7	3 (43)	4 (57)	
Drug type				.71
Small molecule	29	16 (55)	13 (45)	
Biologic	9	6 (67)	3 (33)	
Therapeutic area				.06
Cancer	28	19 (68)	9 (32)	
Noncancer	10	3 (30)	7 (70)	
Orphan drug designation				.56
Yes	35	21 (60)	14 (40)	
No	3	1 (33)	2 (67)	
Confirmatory trial primary outcome				.49
Clinical outcome	13	6 (46)	7 (54)	
Surrogate end point	23	15 (65)	8 (35)	
No data^b^	2	1 (50)	1 (50)	
Supplemental indication				<.001
Yes	20	17 (85)	3 (15)	
No	18	5 (28)	13 (72)	

Abbreviations: FDA, US Food and Drug Administration; NA, not applicable.

^a^ Conversion status as of December 31, 2020.

^b^ For 1 converted and 1 unconverted drug, postapproval clinical trial registrations meeting FDA requirements could not be identified.

**Figure 1.**
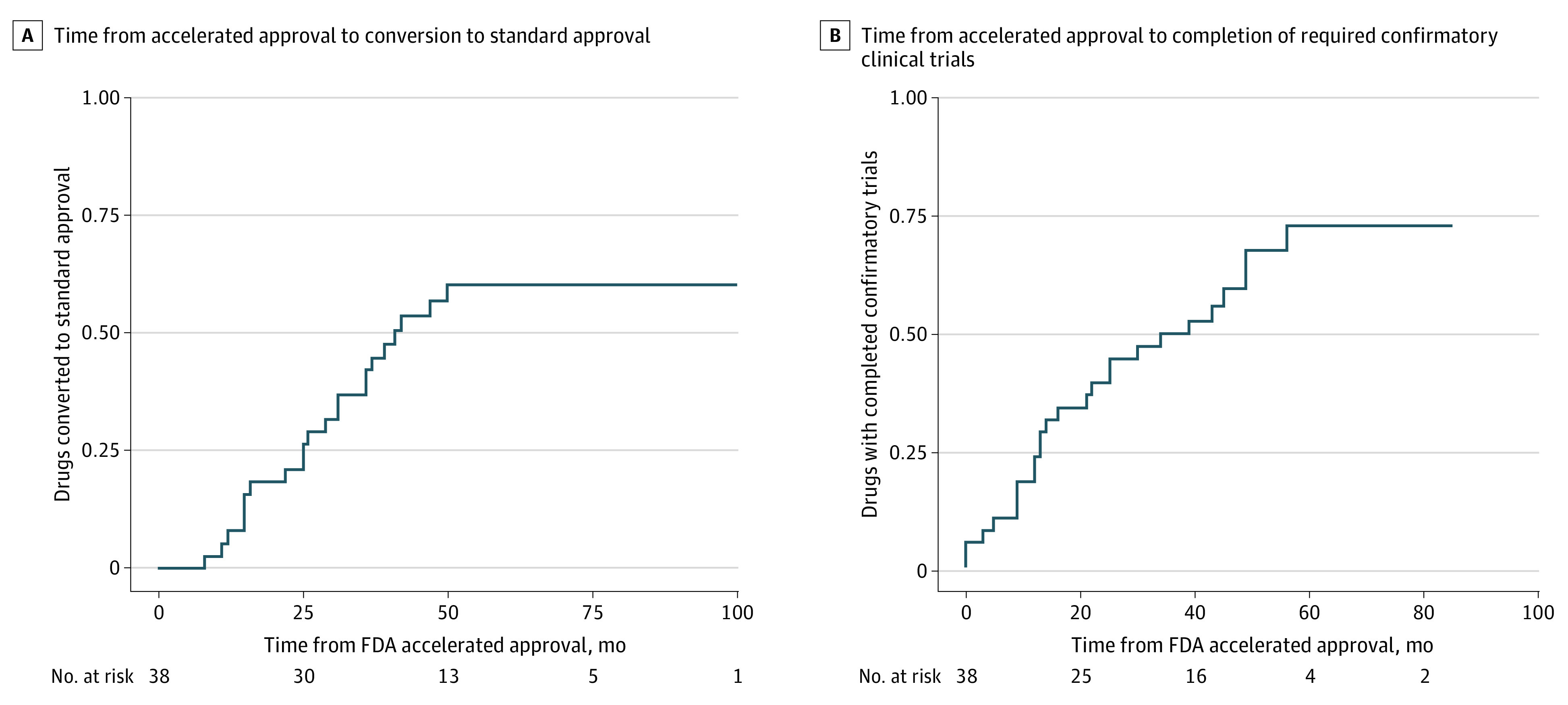
Time From FDA Accelerated Approval Granted Through Confirmatory Trials and Conversion, 2012 to 2017

Through 2020, 22 (58%) of the drugs were converted to standard approval, 16 (42%) were not converted, including 1 original indication that was formally withdrawn by the FDA. Median (IQR) time to conversion was 28 (15-37) months, and conversion increased steadily with time (Figure 1A). The proportion of drugs converted within 3 years of accelerated approval was comparable for drugs approved in 2012 to 2014 vs 2015 to 2017 (7 of 16 [44%] vs 9 of 22 [41%] drugs), with no significant difference (*P* = .41) in conversion status by year of accelerated approval.

Through 2020, the FDA approved 95 supplemental indications for 20 of 38 (53%) drugs that had been granted accelerated approval; 35 (37%) supplemental indications were also granted accelerated approval. Two drugs, pembrolizumab and nivolumab, accounted for one-half (48 of 95 [51%]) of supplemental indication approvals. The median (IQR) time to approval of a drug’s first supplemental indications was 25 (12-32) months. Converted drugs were more likely than unconverted drugs to receive FDA approval for 1 or more supplemental indications (77% vs 19%; *P* < .001).

### Confirmatory Clinical Trials

The FDA postmarketing requirements included 52 confirmatory trials for the 38 drugs granted accelerated approval. At least 1 confirmatory trial was outlined per indication. For 10 indications, 2 trials were required. The median (IQR) time outlined by the FDA for completion of all confirmatory trials for drugs was 49 (24-64) months. The median (IQR) time outlined for submission of all final trial reports was also 49 (30-70) months.

ClinicalTrials.gov registrations were identified for 50 of 52 (96%) confirmatory trials; trial registrations for 2 drugs were not identified. Primary completion of confirmatory trials increased steadily after accelerated approval (Figure 1B). For 21 of 22 drugs that were ultimately converted to standard approval, 34 registered confirmatory trials were identified, including 18 (53%) completed or terminated trials and 16 (47%) trials that remained active or were still recruiting participants through 2020. Among 16 unconverted drugs, confirmatory trials were not completed for 9 (56%) drugs and were completed or terminated without reported results for 3 (19%) drugs. For drugs with confirmatory trials completed through 2020, median (IQR) time to primary completion of all trials was 16 (9-34) months.

**Figure 2.**
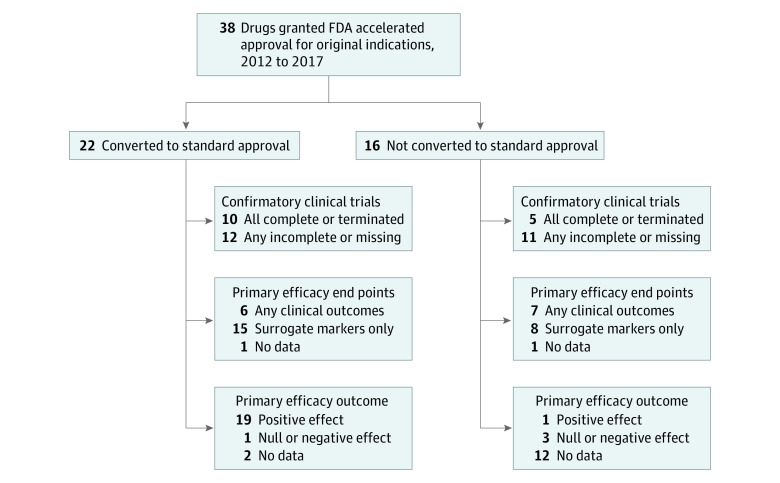
Characteristics and Outcomes of Confirmatory Clinical Trials for Original Indications of Drugs Granted FDA Accelerated Approval, 2012 to 2017

Clinical outcomes, such as overall survival, were assessed as primary efficacy end points in at least 1 confirmatory trial for 13 (34%) drugs, while 23 (61%) used surrogate end points for all trials (Figure 2). Among 22 converted drugs, confirmatory trials demonstrated a positive effect (ie, confirmed benefit) for 19 (86%) drugs, of which 14 were evaluated using surrogate end points. No benefit was found for 1 (5%) drug, and 2 (9%) did not have reported results. Confirmatory trials demonstrated a positive effect for 1 of 16 (6%) unconverted drugs and found no benefit for 3 (19%) of these drugs through 2020.

### CMS Spending

For 37 of the 38 drugs granted FDA accelerated approval, CMS spending was $67.9 billion (Table 2), representing 3% of $2.2 trillion in total spending for the programs from 2012 to 2020. One unconverted drug, deferiprone, was excluded from spending analyses after payments could not be differentiated between multiple products bearing the same name. Median (IQR) overall spending per drug was $329.3 million ($54.4 million-$1.6 billion), and median (IQR) annualized spending was $71.8 ($14.6-$277.7) million.

**Table 2.** Spending by CMS for Drugs Granted FDA Accelerated Approval Before and After Conversion to Standard Approval, 2012 to 2017

Drug characteristic	All drugs	Not converted^a^	Converted to standard approval^b^		
			Total	Before	After
**Overall CMS drug spending (%), $ in millions**					
All drugs	67 873.1	5795.1	62 078.0	11 093.2	50 984.8
Type					
Small molecule	40 883.9 (60)	2894.2 (50)	37 989.6 (61)	4988.3 (45)	33 001.4 (65)
Biologic	26 989.2 (40)	2900.9 (50)	24 088.4 (39)	6104.9 (55)	17 983.4 (35)
Therapeutic area					
Cancer	64 265.8 (95)	3.837.1 (66)	60 428.8 (97)	10 220.7 (92)	50 208.1 (98)
Non-cancer	3607.3 (5)	1958.0 (34)	1649.3 (5)	872.5 (8)	776.8 (2)
Orphan drug designation					
Yes	54 990.8 (81)	2991.4 (52)	51 999.4 (84)	9100.1 (82)	42 899.3 (84)
No	12 882.3 (19)	2803.7 (48)	10 078.6 (16)	1993.1 (18)	8085.5 (16)
Confirmatory trial primary outcome					
Clinical outcome	17 946.3 (26)	4861.4 (84)	13 084.9 (21)	1007.4 (9)	12 077.6 (24)
Surrogate end point	40 286.3 (59)	892.6 (15)	39 393.7 (63)	4123.5 (37)	35 270.2 (69)
No data	9640.5 (14)	41.1 (1)	9599.4 (15)	5962.3 (54)	3.637.1 (7)
Supplemental indication					
Yes	62 664.9 (92)	3316.9 (57)	59 348.0 (96)	10 085.0 (91)	49 263.0 (97)
No	5208.2 (8)	2478.1 (43)	2730.0 (4)	1008.2 (9)	1721.8 (3)
**Annualized CMS drug spending, median (IQR), $ in millions**					
All drugs	71.8 (14.6-277.7)	23.3 (1.2-143.0)	132.4 (27.9-578.4)	35.0 (9.2-150.2)	199.0 (41.3-718.4)
Type					
Small molecule	62.6 (8.6-192.2)	7.9 (0.8-86.3)	132.4 (27.6-332.4)	48.9 (21.8-180.4)	199.0 (42.9-475.9)
Biologic	328.0 (26.7-605.7)	328.0 (175.7-341.6)	319.8 (28.5-1351.3)	9.8 (5.7-25.7)	403.7 (42.8-1746.7)
Therapeutic area					
Cancer	85.3 (21.6-390.5)	23.3 (6.4-162.1)	175.9 (32.6-691.0)	39.4 (13.1-145.7)	276.1 (52.5-859.9)
Noncancer	14.6 (0.3-129.6)	36.4 (0.4-115.1)	14.6 (7.3-146.2)	6.2 (3.1-143.7)	18.0 (9.0-150.3)
Orphan drug designation					
Yes	51.3 (10.6-224.8)	9.3 (0.9-71.8)	89.0 (26.7-496.4)	30.6 (8.6-136.5)	122.0 (38.6-617.8)
No	355.2 (341.6-1041.5)	341.6 (334.8-348.4)	1727.8 (1727.8-1727.8)	956.7 (956.7-956.7)	2156.1 (2156.1-2156.1)
Confirmatory trial primary outcome					
Clinical outcome	71.8 (23.3-277.7)	129.6 (47.6-242.3)	30.3 (17.6-216.8)	8.6 (5.1-25.7)	47.0 (23.1-225.9)
Surrogate end point	67.5 (11.0-253.3)	0.9 (0.3-29.2)	175.9 (42.4-551.0)	45.7 (19.5-145.7)	276.1 (58.7-684.9)
No data	803.1 (404.8-1201.5)	6.4 (6.4-6.4)	1599.9 (1599.9-1599.9)	1431.0 (1431.0-1431.0)	2078.3 (2078.3-2078.3)
Supplemental indication					
Yes	249.2 (48.6-648.3)	328.0 (245.1-341.6)	241.1 (33.9-776.3)	39.4 (15.1-154.8)	315.5 (55.4-967.9)
No	9.3 (0.9-71.8)	7.9 (0.8-54.8)	14.6 (3.1-175.9)	6.2 (0.7-52.2)	18.0 (3.6-276.1)

Abbreviations: CMS, Centers for Medicare & Medicaid Services; FDA, US Food and Drug Administration.

^a^ Excludes 1 drug (deferiprone) for which CMS payments could not be differentiated between multiple products with the same name.

^b^ Prorated based on the month of conversion to standard approval.

Medicare Part D accounted for half ($34.4 billion [51%]) of overall drug spending; Part B covered 12 drugs and accounted for $25.4 billion (37%). Total Medicaid spending was $8.1 billion (12%). Applying estimated rebates reduced Medicare Part D spending from $34.4 billion to $31.1 billion and reduced Medicaid spending from $8.1 billion to $7.5 billion. Applying Medicare Part D’s overall rebate rate of 17.5% to all drugs would reduce its spending to $28.4 billion. For Medicaid, applying the minimum basic rebate rate of 23.1% to all drugs would reduce spending to $6.2 billion.

### Drugs Converted to Standard Approval

Among the 22 converted drugs, median (IQR) time on market was 71 (60-84) months through 2020, and conversion to standard approval occurred a median (IQR) of 28 (15-37) months after accelerated approval. Median (IQR) time from primary completion of all confirmatory trials to conversion was 9 (1-14) months.

Converted drugs accounted for $62.1 billion of $67.9 billion (91%) of CMS spending, including $11.1 billion (18%) before conversion and $51.0 billion (82%) after conversion. Median annualized spending increased more than 5-fold after conversion ($35.0 million vs $199.0 million), with increased spending noted for most drugs across all federal programs covering them (Table 3). Most spending (97%; $60.4 billion) was for cancer treatment products, including for 10 of 11 converted drugs grossing more than $1 billion from 2012 to 2020. The 3 highest grossing drugs, each indicated to treat cancers, accounted for more than half of spending (53%; $32.8 billion) for converted drugs. More than three-fifths of spending (63%; $39.4 billion) for converted drugs was attributable to those evaluated using surrogate end points. Converted drugs with at least 1 confirmatory trial evaluating a primary clinical outcome accounted for one-fifth of spending (21%; $13.1 billion).

**Table 3.** Spending by CMS Before and After Conversion to Standard Approval of Drugs Granted FDA Accelerated Approval, 2012 to 2017

Drug	Approval year	Time on market, mo^a^		CMS spending, $ in millions^b^					
				Medicare Part B		Medicare Part D		Medicaid	
		Before	After	Before	After	Before	After	Before	After
Carfilzomib	2012	42	59	129.7	289.6	0.0	5.1	6.8	20.8
Everolimus	2012	41	59	0.0	0.0	0.7	5.4	4.3	44.2
Omacetaxine	2012	15	82	0.0	0.1	0.6	2.4	0.1	1.1
Ponatinib	2012	47	49	0.0	0.0	27.7	83.1	11.7	38.9
Ibrutinib	2013	8	77	0.0	0.0	246.0	1704.2	11.3	78.0
Pomalidomide	2013	26	68	0.0	0.0	263.2	920.2	12.0	47.6
Blinatumomab	2014	31	41	4.7	20.1	0.6	1.0	5.8	17.5
Ceritinib	2014	36	43	0.0	0.0	19.9	8.5	4.1	1.7
Nivolumab	2014	50	21	1227.9	1790.2	27.7	40.1	175.4	248.0
Olaparib	2014	31	40	0.0	0.0	44.9	228.0	7.3	48.1
Pembrolizumab	2014	15	60	0.0	1943.9	2.2	30.1	2.5	249.3
Alectinib	2015	22	37	0.0	0.0	37.5	87.8	8.2	29.3
Daratumumab	2015	12	49	0.0	696.7	5.0	14.1	3.6	41.2
Deferasirox^c^	2015	37	31	0.0	0.0	132.5	149.6	148.7	133.1
Idarucizumab	2015	29	32	0.0	0.0	0.0	0.0	0.0	0.0
Osimertinib	2015	16	45	0.0	0.0	135.5	546.1	19.3	71.7
Palbociclib	2015	25	45	0.0	0.0	827.4	1838.8	129.3	317.4
Rucaparib	2016	15	32	0.0	0.0	21.3	59.1	5.0	8.7
Venetoclax	2016	25	30	0.0	0.0	58.8	409.8	3.4	18.8
Avelumab	2017	39	6	29.4	51.6	0.4	0.9	0.8	2.9
Brigatinib	2017	36	7	0.0	0.0	12.1	18.1	2.9	4.7
Deferasirox^c^	2017	11	31	0.0	0.0	1.5	4.0	4.7	14.0
Median (IQR)		28 (15-37)	42 (31-57)	0.0 (0.0-0.0)	0.0 (0.0-15.1)	20.6 (0.9-55.3)	35.1 (5.2-208.4)	5.4 (3.4-11.6)	34.1 (10.1-65.8)

Abbreviations: CMS, Centers for Medicare & Medicaid Services; FDA, US Food and Drug Administration.

^a^ Through December 31, 2020.

^b^ Prorated based on the month of conversion to standard approval.

^c^ Two formulations of deferasirox were separately granted accelerated approval, shared confirmatory trials, and were converted on the same date; CMS spending data for these formulations were reported separately.

### Drugs Not Converted to Standard Approval

For the 15 unconverted drugs for which spending data were available, median (IQR) time on market was 55 (47-77) months through 2020. Total CMS spending was $5.8 billion, median (IQR) annualized spending was $23.3 ($1.2 -$143.0) million, and 3 unconverted drugs grossed $1 billion or more through 2020. Unconverted drugs with confirmatory trials evaluating clinical outcomes accounted for $4.9 billion of $5.8 billion (84%) of spending, and those evaluated using surrogate end points accounted for $892.6 million (15%).

### Drugs With Confirmatory Trials Failing to Confirm Benefit

Trials for 1 converted (pembrolizumab) and 3 unconverted (atezolizumab, durvalumab, and olaratumab) original indications failed to confirm benefit for primary efficacy end points. These drugs accounted for $14.0 billion in CMS spending through 2020. The original indication for each unconverted drug was voluntarily withdrawn by sponsors as of June 30, 2021, although atezolizumab and durvalumab remain approved for supplemental indications.^39,40,41,42^

### Sensitivity Analyses

Spending by CMS before approval of the first supplemental indication for each drug was $8.7 billion of $67.9 billion (13%), including $5.2 billion for the 17 drugs with no supplemental indications granted through 2020. After adjusting for the number of supplemental indications granted for each, median (IQR) per-indication spending was $237.9 ($44.7-$603.2) million. Median (IQR) annualized per-indication spending was $47.6 ($9.3-$129.6) million.

## Discussion

For 37 drugs granted accelerated approval by FDA from 2012 to 2017, we identified $67.9 billion spent by CMS, including $11.1 billion for drugs before conversion to standard approval and $5.8 billion for drugs not converted through 2020. More than 90% of spending in this cross section of CMS expenditures occurred for drugs ultimately converted to standard approval, and annualized spending increased after conversion. However, drugs with confirmatory trials evaluating surrogate end points accounted for approximately three-fifths of accelerated approvals, as well as three-fifths of spending. This included many of the highest-grossing drugs granted accelerated approval, nearly all of which were indicated to treat cancer. These findings suggest that drugs that are granted accelerated approval were integrated into clinical practice before confirmation of benefit for original indications, and that therapeutic spending increased after conversion despite continued reliance on surrogate end points in confirmatory trials.

For drugs that were granted accelerated approval from 2012 to 2017, we found that only 34% had a confirmatory trial evaluating a clinical outcome as a primary end point; this finding was comparable to that of previous studies.^10,11^ The accelerated approval pathway was developed to facilitate earlier access to drugs that address the unmet needs of patients with serious conditions, with the expectation that clinical benefit would be confirmed by postapproval trials. However, use of surrogate end points in both preapproval and postapproval trials supporting accelerated approval has been common. For drugs indicated to treat cancer, less than one-third of conversions to standard approval are supported by overall survival data.^12^ Despite the frequent use of surrogate end points in postapproval trials for cancer drugs, most are not strongly correlated with overall survival,^43^ including response rate and progression-free survival.^44^ However, spending increased steadily for cancer drugs after conversion, suggesting that these products are integrated into clinical practice despite persistent uncertainty about whether patients can expect clinical benefit.

For drugs ultimately converted to standard approval, approximately one-quarter of overall spending, $11.1 billion, occurred before conversion. Increased spending after conversion may reflect growing clinical consensus on drugs, which are gradually incorporated into guidelines that influence broader practice.^13^ It may also reflect other factors, however, such as fewer barriers to the promotion of converted drugs.^45,46^ We identified generous deadlines granted by the FDA for reporting confirmatory trial results, a finding comparable to those of previous studies.^7^ Permitted confirmatory trial durations are frequently longer than those for preapproval trials, despite requiring similar times for completion.^8^ For this sample, median time from accelerated approval to conversion was 28 months, just more than one-half of the median time permitted by FDA for confirmatory trial results reporting. These timelines allow drugs to be integrated into clinical practice before their true risk and benefit profiles are known.

Drug indications may also remain authorized for years after completion of postapproval clinical trials that fail to confirm benefit—observed for 4 therapeutic indications in this study sample—potentially resulting in substantial spending for drugs not supported by clinical evidence.^47^ Although supplemental indications may account for a portion of ongoing therapeutic spending, these additional indications are also frequently approved on the basis of limited clinical evidence.^48,49^ Prolonged study periods for confirmatory trial completion and the pervasive use of surrogate end points may lead to unclear use of drugs for most approved indications after FDA approval.^22^ Clinical uncertainty exposes patients, as well as payers such as CMS, to substantial risk, as these drugs may be covered for years before the FDA or sponsors withdraw their ineffective indications. By that time, these drugs are likely to be integrated into clinical practice and are often approved for multiple supplemental indications.

By estimating expenditure during the years after accelerated approval of new drugs, this study reinforces concerns over growing costs associated with Medicaid’s mandatory coverage of FDA-approved drugs and Medicare’s mandatory coverage of specific drug classes, including cancer drugs.^50^ This study also suggests opportunities for collaboration between CMS and the FDA to ensure that therapeutic spending is supported by strong clinical evidence. Given the substantial revenue for sponsors from federal payers, the FDA should require evaluation of clinical outcomes in trials supporting conversion to standard approval.

Several other proposals have been made to address ongoing problems associated with accelerated approval.^51,52^ Unconverted products could be excluded from coverage mandates,^53^ potentially incentivizing use of the pathway for drugs with promising preapproval evidence of benefit, as well as timely completion of confirmatory trials. In addition, CMS could connect rebate rates to the availability of evidence supporting therapeutic effectiveness on clinical outcomes^54^ or directly to patient outcomes,^55^ and could withhold or recollect payments when confirmatory trials do not confirm benefit.^56^ Alternatively, empowering CMS to decline coverage of ineffective drugs, similar to cost-effectiveness determinations by other national drugs payers, could reduce expenditure on low-value care, albeit at the risk of slower drug development and less use of expedited review pathways.^57^ Finally, the FDA should continue to review drug sponsors compliance with confirmatory trial requirements, enforce its deadlines, and quickly withdraw market authorization for indications that fail to confirm clinical benefit.

### Limitations

This study had several limitations. First, we estimated spending using the CMS Drug Spending Dashboards, which do not include patient-level data, such as disease severity or treatment history, which are required to index spending to specific therapeutic indications. As a result, the study estimates were unable to account for the proportion of spending attributable to original, supplemental, and off-label indications. Second, we did not account for changes in overall CMS enrollment during the study period. Moreover, the study estimates do not include spending by Medicare Advantage plans, which in 2020 enrolled 39% of Medicare beneficiaries,^58^ thereby underestimating overall Medicare spending. In addition, we did not calculate beneficiary out-of-pocket costs. These estimates represent a cross section of federal spending during the study period, rather than a longitudinal analysis of therapeutic use and costs. Third, CMS does not report individual drug rebates, including inflation-based rebates paid to Medicaid, which are not publicly available and accounted for 54% of payments by sponsors to the program in 2012.^59^ Rebate estimation remains a methodological challenge for analyses of CMS spending.^60^ We applied overall program rebate rates when drug or drug class estimates were not available; it is possible that this approach underestimated Medicaid and overestimated Medicare rebates.^61^ Finally, we did not account for variation between drugs in the time required to stage postapproval clinical trials, and it is possible that the FDA will consider additional evidence sources that do evaluate clinical outcomes when determining whether to convert drugs to standard approval. However, postapproval trial durations are typically comparable with pivotal trial durations,^8^ and in the absence of consistently available public information on the FDA’s rationale for conversion determinations, it is not possible to assess how trial logistics or complementary sources of clinical evidence may influence conversion.

## Conclusions

This cross-sectional study found that most drugs granted FDA accelerated approval for original indications from 2012 to 2017 lacked confirmatory trials that evaluated clinical outcomes to support conversion to standard approval. A substantial portion of CMS spending was for drugs with confirmatory trials evaluating surrogate end points, and automatic coverage mandates led to substantial spending despite residual uncertainty regarding clinical benefit to patients. The accelerated approval pathway is intended to facilitate access to drugs that address unmet medical needs. However, persistent evidentiary gaps should prompt payers to limit spending on promising drugs with unproven benefits.
